# (*Z*)-2-Phenyl-3-pivaloyl-1,1-dipropyl­guanidine

**DOI:** 10.1107/S160053680902978X

**Published:** 2009-08-08

**Authors:** Muhammad Said, Ghulam Murtaza, Eva Freisinger, Saeed Anwar, Abdur Rauf

**Affiliations:** a Department of Chemistry, Quaid-i-Azam University, Islamabad 45320, Pakistan; b University of Zürich, Institute of Inorganic Chemistry, Winterthurerstrasse 190, 8057-Zürich, Switzerland; c Department of Chemistry, Abdul Wali Khan University, Mardan, Pakistan; d Department of Chemistry, Islamia University of Bahawalpur, Pakistan

## Abstract

In the title compound, C_18_H_29_N_3_O, a polysubstituted guanidine, the torsion angles indicate that the guanidine unit and the carbonyl group are almost perpendicular to one another [O—C—N—C= −7.40 (18), C—N—C—N= −97.21 (15) and 86.41 (13)°]. The crystal packing is stablized by inter­molecular N—H⋯O hydrogen bonds, which link the mol­ecules into a chain.

## Related literature

For the biological and chemical properties of guanidine derivatives, see: Ohara *et al.* (2007 ▶); Berlinck (2002 ▶); Ma *et al.* (2008 ▶); Brzozowski *et al.* (2007 ▶); Gomez *et al.* (2000 ▶); Kovacevic & Maksic (2001 ▶); Ishikawa & Isobe (2002 ▶); Rauf *et al.* (2009 ▶). For related structures, see: Cunha *et al.* (2005 ▶); Murtaza *et al.* (2007 ▶, 2008 ▶, 2009 ▶).
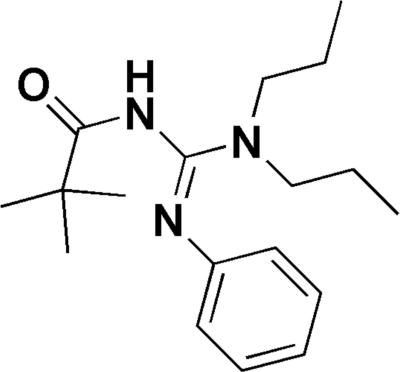


## Experimental

### 

#### Crystal data

C_18_H_29_N_3_O
*M* *_r_* = 303.44
Orthorhombic, 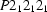

*a* = 9.898 (5) Å
*b* = 12.648 (5) Å
*c* = 15.126 (5) Å
*V* = 1893.6 (14) Å^3^
*Z* = 4
Mo *K*α radiation
μ = 0.07 mm^−1^
*T* = 183 K
0.42 × 0.42 × 0.32 mm

#### Data collection

Oxford diffraction Xcalibur R diffractometer
Absorption correction: multi-scan (*CrysAlis RED*; Oxford Diffraction, 2006 ▶) *T*_min_ = 0.973, *T*_max_ = 0.979
31051 measured reflections
7202 independent reflections
5107 reflections with *I* > 2σ(*I*)
*R*_int_ = 0.029

#### Refinement

*R*[*F* ^2^ > 2σ(*F* ^2^)] = 0.056
*wR*(*F* ^2^) = 0.134
*S* = 0.97
7202 reflections
211 parameters
H atoms treated by a mixture of independent and constrained refinement
Δρ_max_ = 0.30 e Å^−3^
Δρ_min_ = −0.18 e Å^−3^
Absolute structure: Flack (1983 ▶),3174 Friedel pairs
Flack parameter: 0.20 (12)

### 

Data collection: *CrysAlis CCD* (Oxford Diffraction, 2006 ▶); cell refinement: *CrysAlis RED* (Oxford Diffraction, 2006 ▶); data reduction: *CrysAlis RED*; program(s) used to solve structure: *SHELXS97* (Sheldrick, 2008 ▶); program(s) used to refine structure: *SHELXL97* (Sheldrick, 2008 ▶); molecular graphics: *ORTEP-3* (Farrugia, 1997 ▶); software used to prepare material for publication: *SHELXL97*.

## Supplementary Material

Crystal structure: contains datablocks I, global. DOI: 10.1107/S160053680902978X/su2130sup1.cif

Structure factors: contains datablocks I. DOI: 10.1107/S160053680902978X/su2130Isup2.hkl

Additional supplementary materials:  crystallographic information; 3D view; checkCIF report

## Figures and Tables

**Table 1:** Hydrogen-bond geometry (Å, °)

*D*—H⋯*A*	*D*—H	H⋯*A*	*D*⋯*A*	*D*—H⋯*A*
N1—H1⋯O1^i^	0.837 (17)	2.01 (2)	2.830 (2)	165 (1)

Symmetry code: (i) 
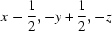
.

**Fig. 1.**
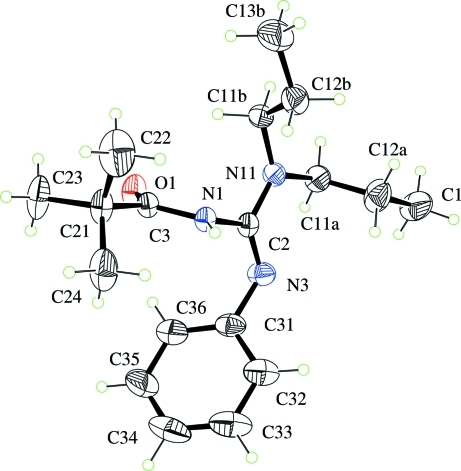
Molecular structure of of the title compound, showing the atom numbering scheme and displacement ellipsoids drawn at the 50% probability level.

**Fig. 2.**
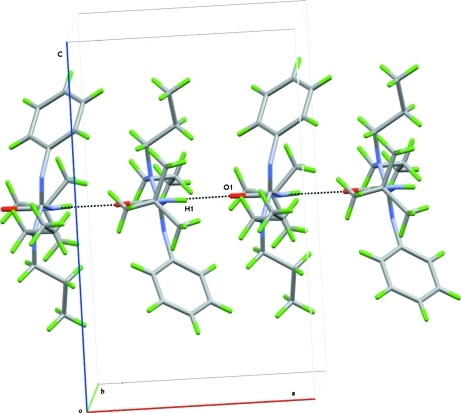
A view of the formation of the N—H···O intermolecular hydrogen bonded chain of molecules of the title compound (see Table 1 for details).

## supplementary crystallographic information

### Comment

The guanidinium moiety is present in diverse biologically active natural substances as well as in a number of medicinal compounds (Berlinck, 2002). Polysubstituted guanidines had received considerable interest as DNA binders (Ohara *et al.*, 2007) and as anticancer agents (Ma *et al.*, 2008; Brzozowski *et al.*, 2007). In addition to their biological role, guanidine derivatives are widely utilized in synthetic organic chemistry, due to their high catalytic potential (Gomez *et al.*, 2000; Kovacevic & Maksic, 2001). Due to their high proton affinity, guanidines can be considered as super-bases (Ishikawa & Isobe, 2002).

The title compound (Fig. 1) is a typical tetra-substituted guanidine with normal geometric parameters (Cunha *et al.*, 2005; Murtaza *et al.*, 2007, 2008, 2009). The C3—O1 bond shows the expected full double bond character [1.2243 (15) Å ]. The short value for the C2—N3 bond length [1.2812 (16) Å] also shows double bond character, while the values for the C2—N11, C2—N1, and C3—N1 bond lengths [1.3548 (15), 1.4348 (15) and 1.3510 (14) Å, respectively] indicate partial double bond character. The dihedral angles between the guanidine mean plane [C(2)/N(1)/N(3)/N(11)] and the phenyl ring [C31–C36] is 67.96 (10)°. The carbonyl group [C3═O1] is almost perpendicular the guanidine moiety mean plane, as reflected by torsion angles O1—C3—N1—C2= -7.40 (18)°, C3—N1—C2—N3= -97.21 (15)°, and C3—N1—C2—N11= 86.41 (13)°. This is probably due to the absence of intramolecular N—H···O hydrogen bonding forming a six-membered ring, which is commonly observed in this class of compounds (Cunha *et al.*, 2005).

The crystal packing of the title compound shows intermolecular N—H···O hydrogen bonds, which link the molecules into a continuous chain (Fig. 2).

### Experimental

1-phenyl-3-(pivaloyl)thiourea (0.236 g, 1 mmol) [Rauf *et al.*, 2009], dissolved in 10 ml of DMF, was placed in a two neck round bottom flask. Dipropylamine (0.14 g, 1 mmol) and triethylamine (0.28 ml, 2 mmol) were added and the mixture was stirred well at a temperature below 278 K. Mercuric chloride (0.272 g, 1 mmol) was then added and the mixture was stirred vigorously for 20 h. The progress of the reaction was monitored by TLC, untill the completion of reaction. When all the thiourea had been consumed, 20 ml of CH_2_Cl_2_ was added and the suspension was filtered through a cintered glass funnel to remove residual HgS, formed as a byproduct during the reaction. The solvent was then evaporated under reduced pressure and the residue dissolved in 20 ml of CH_2_Cl_2_. Other byproducts were extracted out with water (4×30 ml). The organic phase was dried over anhydrous MgSO_4_ and then filtered. The solvent was evaporated and the product was further purified by column chromatography. The target guanidine was recrystallized using ethanol.

### Refinement

The NH H-atom was located in a different electron-density map and freely refined: N-H = 0.837 (17) Å. The C-bound H-atoms were included in calculated positions and treated as riding atoms: C—H = 0.95 - 0.98 Å with *U*_iso_(H) = k × U_eq_(C), where k = 1.2 for H-aromatic, and 1.5 for H-methyl.

### Crystal data

**Table:**

C_18_H_29_N_3_O	*F*(000) = 664
*M**_r_* = 303.44	*D*_x_ = 1.064 Mg m^−^^3^
Orthorhombic, *P*2_1_2_1_2_1_	Mo *K*α radiation, λ = 0.71073 Å
Hall symbol: P 2ac 2ab	Cell parameters from 31051 reflections
*a* = 9.898 (5) Å	θ = 2.5–33.1°
*b* = 12.648 (5) Å	µ = 0.07 mm^−^^1^
*c* = 15.126 (5) Å	*T* = 183 K
*V* = 1893.6 (14) Å^3^	Block, colourless
*Z* = 4	0.42 × 0.42 × 0.32 mm

### Data collection

**Table:**

Oxford diffraction Xcalibur R diffractometer	7202 independent reflections
Radiation source: Enhance (Mo) X-ray Source	5107 reflections with *I* > 2σ(*I*)
graphite	*R*_int_ = 0.029
Detector resolution: 10.4498 pixels mm^-1^	θ_max_ = 33.1°, θ_min_ = 2.5°
Profile data from ω scans	*h* = −14→15
Absorption correction: multi-scan (*CrysAlis RED*; Oxford Diffraction, 2006)	*k* = −19→19
*T*_min_ = 0.973, *T*_max_ = 0.979	*l* = −23→23
31051 measured reflections	

### Refinement

**Table:**

Refinement on *F*^2^	Secondary atom site location: difference Fourier map
Least-squares matrix: full	Hydrogen site location: inferred from neighbouring sites
*R*[*F*^2^ > 2σ(*F*^2^)] = 0.056	H atoms treated by a mixture of independent and constrained refinement
*wR*(*F*^2^) = 0.134	*w* = 1/[σ^2^(*F*_o_^2^) + (0.0786*P*)^2^] where *P* = (*F*_o_^2^ + 2*F*_c_^2^)/3
*S* = 0.97	(Δ/σ)_max_ < 0.001
7202 reflections	Δρ_max_ = 0.30 e Å^−^^3^
211 parameters	Δρ_min_ = −0.18 e Å^−^^3^
0 restraints	Absolute structure: Flack (1983), 3174 Friedel pairs
Primary atom site location: structure-invariant direct methods	Flack parameter: 0.20 (12)

### Special details

**Table:**

Experimental. Empirical absorption correction using spherical harmonics, implemented in SCALE3 ABSPACK scaling algorithm CrysAlis RED (Oxford Diffraction, 2006)
Geometry. All e.s.d.'s (except the e.s.d. in the dihedral angle between two l.s. planes) are estimated using the full covariance matrix. The cell e.s.d.'s are taken into account individually in the estimation of e.s.d.'s in distances, angles and torsion angles; correlations between e.s.d.'s in cell parameters are only used when they are defined by crystal symmetry. An approximate (isotropic) treatment of cell e.s.d.'s is used for estimating e.s.d.'s involving l.s. planes.
Refinement. Refinement of *F*^2^ against ALL reflections. The weighted *R*-factor *wR* and goodness of fit *S* are based on *F*^2^, conventional *R*-factors *R* are based on *F*, with *F* set to zero for negative *F*^2^. The threshold expression of *F*^2^ > σ(*F*^2^) is used only for calculating *R*-factors(gt) *etc*. and is not relevant to the choice of reflections for refinement. *R*-factors based on *F*^2^ are statistically about twice as large as those based on *F*, and *R*- factors based on ALL data will be even larger.

### Fractional atomic coordinates and isotropic or equivalent isotropic displacement parameters (Å^2^)

**Table:**

	*x*	*y*	*z*	*U*_iso_*/*U*_eq_	
N1	0.64742 (9)	0.28579 (7)	0.00092 (7)	0.02445 (18)	
H1	0.5657 (17)	0.2689 (11)	0.0027 (9)	0.031 (3)*	
N3	0.67372 (13)	0.43416 (8)	0.09671 (7)	0.0375 (3)	
N11	0.71989 (11)	0.45147 (7)	−0.05199 (7)	0.0299 (2)	
C11A	0.74991 (15)	0.56368 (9)	−0.03790 (10)	0.0386 (3)	
H11A	0.8100	0.5889	−0.0858	0.080*	
H11B	0.7988	0.5719	0.0188	0.080*	
C12A	0.62283 (18)	0.63189 (11)	−0.03603 (12)	0.0520 (4)	
H12A	0.5797	0.6314	−0.0951	0.080*	
H12B	0.5578	0.6023	0.0070	0.080*	
C13A	0.6579 (2)	0.74502 (11)	−0.01020 (13)	0.0614 (5)	
H13A	0.7048	0.7450	0.0469	0.0803 (17)*	
H13B	0.5748	0.7867	−0.0055	0.0803 (17)*	
H13C	0.7166	0.7761	−0.0554	0.0803 (17)*	
C11B	0.72858 (13)	0.40828 (10)	−0.14124 (8)	0.0331 (3)	
H11C	0.7649	0.3355	−0.1376	0.080*	
H11D	0.7938	0.4511	−0.1755	0.080*	
C12B	0.59584 (17)	0.40521 (13)	−0.19124 (10)	0.0459 (3)	
H12C	0.5610	0.4780	−0.1986	0.080*	
H12D	0.5286	0.3644	−0.1569	0.080*	
C13B	0.6147 (2)	0.35420 (16)	−0.28203 (11)	0.0632 (5)	
H13D	0.6810	0.3948	−0.3161	0.0803 (17)*	
H13E	0.5282	0.3536	−0.3136	0.0803 (17)*	
H13F	0.6471	0.2815	−0.2746	0.0803 (17)*	
O1	0.86233 (8)	0.23044 (7)	0.00147 (7)	0.0390 (2)	
C2	0.68297 (11)	0.39354 (8)	0.01936 (8)	0.0265 (2)	
C3	0.74191 (11)	0.20881 (8)	−0.00239 (8)	0.0271 (2)	
C21	0.68941 (12)	0.09564 (9)	−0.01267 (10)	0.0366 (3)	
C22	0.6283 (2)	0.08507 (15)	−0.10465 (14)	0.0698 (6)	
H22A	0.5525	0.1344	−0.1107	0.0803 (17)*	
H22B	0.5960	0.0126	−0.1133	0.0803 (17)*	
H22C	0.6971	0.1015	−0.1491	0.0803 (17)*	
C23	0.80917 (14)	0.02055 (10)	−0.00221 (14)	0.0550 (4)	
H23A	0.8782	0.0377	−0.0464	0.0803 (17)*	
H23B	0.7787	−0.0525	−0.0107	0.0803 (17)*	
H23C	0.8475	0.0283	0.0572	0.0803 (17)*	
C24	0.58315 (17)	0.07004 (12)	0.05771 (15)	0.0597 (5)	
H24A	0.6207	0.0838	0.1166	0.0803 (17)*	
H24B	0.5574	−0.0046	0.0532	0.0803 (17)*	
H24C	0.5033	0.1145	0.0484	0.0803 (17)*	
C31	0.64035 (15)	0.36809 (11)	0.16887 (8)	0.0377 (3)	
C32	0.51984 (17)	0.38671 (15)	0.21422 (10)	0.0513 (4)	
H32	0.4640	0.4448	0.1983	0.070 (3)*	
C33	0.4819 (2)	0.31964 (17)	0.28296 (11)	0.0613 (5)	
H33	0.3985	0.3310	0.3124	0.070 (3)*	
C34	0.5638 (2)	0.23707 (16)	0.30876 (11)	0.0648 (5)	
H34	0.5363	0.1906	0.3547	0.070 (3)*	
C35	0.6853 (2)	0.22300 (16)	0.26725 (10)	0.0650 (5)	
H35	0.7439	0.1679	0.2860	0.070 (3)*	
C36	0.72416 (19)	0.28850 (15)	0.19788 (11)	0.0530 (4)	
H36	0.8093	0.2782	0.1703	0.070 (3)*	

### Atomic displacement parameters (Å^2^)

**Table:**

	*U*^11^	*U*^22^	*U*^33^	*U*^12^	*U*^13^	*U*^23^
N1	0.0163 (4)	0.0234 (4)	0.0336 (5)	−0.0025 (3)	0.0004 (4)	0.0011 (4)
N3	0.0474 (7)	0.0315 (5)	0.0335 (5)	−0.0081 (5)	0.0063 (5)	−0.0019 (4)
N11	0.0322 (5)	0.0245 (4)	0.0329 (5)	−0.0028 (4)	0.0041 (4)	0.0026 (4)
C11A	0.0450 (8)	0.0240 (5)	0.0469 (7)	−0.0079 (5)	0.0106 (6)	0.0021 (5)
C12A	0.0585 (10)	0.0288 (6)	0.0687 (10)	0.0005 (6)	0.0091 (8)	−0.0044 (6)
C13A	0.0930 (13)	0.0275 (6)	0.0637 (10)	−0.0057 (7)	0.0275 (10)	−0.0062 (6)
C11B	0.0347 (6)	0.0330 (5)	0.0316 (6)	−0.0006 (5)	0.0067 (5)	0.0056 (5)
C12B	0.0480 (8)	0.0471 (8)	0.0427 (7)	0.0090 (6)	−0.0081 (7)	0.0005 (7)
C13B	0.0798 (13)	0.0689 (11)	0.0409 (9)	0.0042 (10)	−0.0133 (9)	−0.0021 (8)
O1	0.0181 (4)	0.0343 (4)	0.0647 (6)	−0.0013 (3)	0.0010 (4)	0.0017 (5)
C2	0.0207 (5)	0.0247 (5)	0.0340 (6)	−0.0016 (4)	0.0005 (4)	0.0011 (4)
C3	0.0194 (5)	0.0260 (4)	0.0358 (6)	−0.0018 (4)	0.0006 (5)	0.0030 (5)
C21	0.0234 (5)	0.0243 (5)	0.0621 (8)	−0.0007 (4)	−0.0032 (6)	0.0000 (5)
C22	0.0741 (13)	0.0471 (9)	0.0882 (14)	−0.0103 (8)	−0.0347 (11)	−0.0170 (9)
C23	0.0337 (7)	0.0287 (6)	0.1028 (14)	0.0057 (5)	−0.0018 (9)	−0.0006 (8)
C24	0.0383 (8)	0.0322 (7)	0.1086 (15)	−0.0060 (6)	0.0181 (9)	0.0162 (8)
C31	0.0472 (8)	0.0387 (7)	0.0271 (6)	−0.0112 (6)	0.0022 (5)	−0.0056 (5)
C32	0.0471 (9)	0.0683 (11)	0.0384 (8)	−0.0075 (8)	0.0036 (7)	0.0031 (7)
C33	0.0550 (10)	0.0903 (14)	0.0387 (8)	−0.0193 (10)	0.0083 (8)	0.0046 (8)
C34	0.0904 (14)	0.0744 (12)	0.0296 (7)	−0.0249 (11)	0.0002 (9)	0.0103 (8)
C35	0.0958 (15)	0.0657 (11)	0.0334 (7)	0.0116 (11)	−0.0007 (9)	0.0094 (7)
C36	0.0615 (10)	0.0613 (9)	0.0364 (7)	0.0057 (8)	0.0063 (7)	0.0039 (7)

### Geometric parameters (Å, °)

**Table:**

N1—C3	1.3510 (14)	O1—C3	1.2243 (15)
N1—C2	1.4348 (15)	C3—C21	1.5307 (16)
N1—H1	0.837 (17)	C21—C22	1.522 (2)
N3—C2	1.2812 (16)	C21—C23	1.5270 (18)
N3—C31	1.4140 (17)	C21—C24	1.532 (2)
N11—C2	1.3548 (15)	C22—H22A	0.9800
N11—C11B	1.4590 (17)	C22—H22B	0.9800
N11—C11A	1.4656 (16)	C22—H22C	0.9800
C11A—C12A	1.526 (2)	C23—H23A	0.9800
C11A—H11A	0.9900	C23—H23B	0.9800
C11A—H11B	0.9900	C23—H23C	0.9800
C12A—C13A	1.523 (2)	C24—H24A	0.9800
C12A—H12A	0.9900	C24—H24B	0.9800
C12A—H12B	0.9900	C24—H24C	0.9800
C13A—H13A	0.9800	C31—C36	1.376 (2)
C13A—H13B	0.9800	C31—C32	1.396 (2)
C13A—H13C	0.9800	C32—C33	1.394 (2)
C11B—C12B	1.516 (2)	C32—H32	0.9500
C11B—H11C	0.9900	C33—C34	1.378 (3)
C11B—H11D	0.9900	C33—H33	0.9500
C12B—C13B	1.529 (2)	C34—C35	1.369 (3)
C12B—H12C	0.9900	C34—H34	0.9500
C12B—H12D	0.9900	C35—C36	1.391 (3)
C13B—H13D	0.9800	C35—H35	0.9500
C13B—H13E	0.9800	C36—H36	0.9500
C13B—H13F	0.9800		
			
C3—N1—C2	121.47 (9)	O1—C3—N1	120.74 (10)
C3—N1—H1	119.1 (9)	O1—C3—C21	122.97 (10)
C2—N1—H1	118.2 (9)	N1—C3—C21	116.28 (10)
C2—N3—C31	119.01 (10)	C22—C21—C23	110.35 (15)
C2—N11—C11B	123.42 (10)	C22—C21—C24	110.14 (15)
C2—N11—C11A	117.56 (10)	C23—C21—C24	109.27 (13)
C11B—N11—C11A	119.01 (10)	C22—C21—C3	108.02 (12)
N11—C11A—C12A	112.54 (12)	C23—C21—C3	107.92 (10)
N11—C11A—H11A	109.1	C24—C21—C3	111.11 (12)
C12A—C11A—H11A	109.1	C21—C22—H22A	109.5
N11—C11A—H11B	109.1	C21—C22—H22B	109.5
C12A—C11A—H11B	109.1	H22A—C22—H22B	109.5
H11A—C11A—H11B	107.8	C21—C22—H22C	109.5
C13A—C12A—C11A	110.38 (15)	H22A—C22—H22C	109.5
C13A—C12A—H12A	109.6	H22B—C22—H22C	109.5
C11A—C12A—H12A	109.6	C21—C23—H23A	109.5
C13A—C12A—H12B	109.6	C21—C23—H23B	109.5
C11A—C12A—H12B	109.6	H23A—C23—H23B	109.5
H12A—C12A—H12B	108.1	C21—C23—H23C	109.5
C12A—C13A—H13A	109.5	H23A—C23—H23C	109.5
C12A—C13A—H13B	109.5	H23B—C23—H23C	109.5
H13A—C13A—H13B	109.5	C21—C24—H24A	109.5
C12A—C13A—H13C	109.5	C21—C24—H24B	109.5
H13A—C13A—H13C	109.5	H24A—C24—H24B	109.5
H13B—C13A—H13C	109.5	C21—C24—H24C	109.5
N11—C11B—C12B	114.80 (11)	H24A—C24—H24C	109.5
N11—C11B—H11C	108.6	H24B—C24—H24C	109.5
C12B—C11B—H11C	108.6	C36—C31—C32	118.80 (14)
N11—C11B—H11D	108.6	C36—C31—N3	122.50 (14)
C12B—C11B—H11D	108.6	C32—C31—N3	118.67 (14)
H11C—C11B—H11D	107.5	C33—C32—C31	119.64 (17)
C11B—C12B—C13B	110.64 (14)	C33—C32—H32	120.2
C11B—C12B—H12C	109.5	C31—C32—H32	120.2
C13B—C12B—H12C	109.5	C34—C33—C32	120.91 (18)
C11B—C12B—H12D	109.5	C34—C33—H33	119.5
C13B—C12B—H12D	109.5	C32—C33—H33	119.5
H12C—C12B—H12D	108.1	C35—C34—C33	119.04 (17)
C12B—C13B—H13D	109.5	C35—C34—H34	120.5
C12B—C13B—H13E	109.5	C33—C34—H34	120.5
H13D—C13B—H13E	109.5	C34—C35—C36	120.78 (19)
C12B—C13B—H13F	109.5	C34—C35—H35	119.6
H13D—C13B—H13F	109.5	C36—C35—H35	119.6
H13E—C13B—H13F	109.5	C31—C36—C35	120.62 (17)
N3—C2—N11	122.01 (10)	C31—C36—H36	119.7
N3—C2—N1	122.72 (10)	C35—C36—H36	119.7
N11—C2—N1	115.16 (10)		
			
C2—N11—C11A—C12A	−82.33 (15)	O1—C3—C21—C22	−110.60 (16)
C11B—N11—C11A—C12A	96.56 (14)	N1—C3—C21—C22	68.37 (16)
N11—C11A—C12A—C13A	173.14 (13)	O1—C3—C21—C23	8.71 (19)
C2—N11—C11B—C12B	83.80 (15)	N1—C3—C21—C23	−172.32 (13)
C11A—N11—C11B—C12B	−95.02 (14)	O1—C3—C21—C24	128.49 (15)
N11—C11B—C12B—C13B	−177.43 (13)	N1—C3—C21—C24	−52.54 (16)
C31—N3—C2—N11	−177.27 (12)	C2—N3—C31—C36	65.47 (19)
C31—N3—C2—N1	6.59 (19)	C2—N3—C31—C32	−116.74 (15)
C11B—N11—C2—N3	−178.62 (12)	C36—C31—C32—C33	−5.0 (2)
C11A—N11—C2—N3	0.21 (18)	N3—C31—C32—C33	177.09 (14)
C11B—N11—C2—N1	−2.21 (16)	C31—C32—C33—C34	2.1 (3)
C11A—N11—C2—N1	176.62 (10)	C32—C33—C34—C35	1.6 (3)
C3—N1—C2—N3	−97.25 (15)	C33—C34—C35—C36	−2.3 (3)
C3—N1—C2—N11	86.36 (13)	C32—C31—C36—C35	4.4 (2)
C2—N1—C3—O1	−7.40 (18)	N3—C31—C36—C35	−177.80 (15)
C2—N1—C3—C21	173.61 (12)	C34—C35—C36—C31	−0.8 (3)

### Hydrogen-bond geometry (Å, °)

**Table:**

*D*—H···*A*	*D*—H	H···*A*	*D*···*A*	*D*—H···*A*
N1—H1···O1^i^	0.837 (17)	2.01 (2)	2.830 (2)	165 (1)

Symmetry codes: (i) *x*−1/2, −*y*+1/2, −*z*.
